# Legal Intelligence

**Published:** 1900-02-24

**Authors:** 


### LEGAL INTELLIGENCE.

### APHASIA AND TESTAMENTARY CAPACITY.

A very curious will caseinvolving the question of the testamentary capacity of a person suffering from aphasia was decided in the Probate Division of the High Court on the 12th inst. Probate was claimed of the will of the late Marion Edith Moore, who died on August 26th, 1899, the will being dated August 4th of the same year. Against this it was alleged that the will was not duly executed, and was not in accordance with the deceased's instructions, and further that the deceased was not of sound disposing mind. It appeared from the statement made by counsel that the deceased had a stroke of paralysis in July, 1898, which left her the victim of aphasia, and her medical attendant soon afterwards intimated to her that it would be desirable for her to make her will, giving it as his opinion that although his patient had lost the power of coining her thoughts into words her wishes could be satisfactorily ascertained, and he suggested that a number of cards with the various items of her property indicated upon them should be given to the patient together with another pack with the names of her several relatives.

Dr. James Edmunds gave evidence that he attended Miss Moore on July 10th last. She was then suffering from a slight stroke of paralysis and her speech was affected. She knew him perfectly from the first, and could express her assent or dissent, although she was unable to name things, having lost the power of coining her thoughts into words. After consultation with Dr. Lionel Beale he suggested that she ought to make her will, and she assented. He then suggested the arrangement with the cards, using two packs, one containing the items of her property and the other the names of all her relatives. According to the *Times* report evidence was given that "The solicitor put down a card bearing the name of the Ballycoby estate, and the testatrix after looking through her 'pack of relatives' played a name. The solicitor then turned the 'trick.' The rest of the property was then similarly disposed of." In regard to the choice of an executor she did not approve of any of the names, but ultimately threw down a card bearing the name of Mr. John Arthur Moore, the plaintiff in this case. The following day, the will having been prepared in accordance with the pairing of the cards, she executed it by making her mark. She was able to read the newspapers and to say her prayers; her mind was quite clear. The only difficulty there was in ascertaining her wishes was as to names which were not on the cards. She, however, appeared quite satisfied with the names she had before her.

Evidence was further given to the effect that no names of friends—none but those of relatives—appeared on the cards. In giving judgment the President said that, although the case was a peculiar one, nothing could have been more fair and skilful than the manner of ascertaining her real wishes. Had it appeared, however, that all her relatives had not been named on the cards, and had there been anything to lead the Court to suppose that all her wishes had not been carried out, the matter might have been different. It was quite true that in this case it would seem that the testatrix gave indications as to her wish to have two executors, though she was unable to communicate to her solicitor who the second should be. However, when the will was read over to her, as it was carefully afterwards, she showed no sign of any disapproval, which she undoubtedly could have done had she wished to. The will would therefore be admitted to probate.

The interest of this case lies in the fact that the will was admitted to probate, even though the "cards" contained only a selection of names, the names, that is, of relatives only, and did not contain the names of friends or of all possible or probable legatees, such as institutions, to which she might have wished to leave a bequest; and, secondly, that there admittedly had been a difficulty which had not been surmounted in regard to the selection of the executor. Both these difficulties seem, from the language of the judge, to have been held to have been overcome by the fact that when the will was read over she expressed no disapproval. But in regard to this point, which is a very important one, it must be remembered that the Judge added that she certainly could have expressed such disapproval if she wished to do so. It must never be assumed that absence of disapproval means consent in the drawing up of the will of an aphasic. Writing on this subject in "Allbutt's System of Medicine," Dr. Savage says: "Lawyers and some juries have got a vague, general idea that a person may have speech defect without any real intellectual loss, and it will be useless to refer to aphasia as a sign of mental defect if a fairly reasonable will be propounded. There is, in my opinion, always some mental failure with the aphasia, but this varies very greatly. . . . On the other hand, lawyers may attempt to endeavour to establish capacity when a testator affected with aphasia can express himself as satisfied with what is read to him; yet in many cases consent is only automatic; a mere 'yes' to a question does not necessarily import understanding."

It often happens that aphasia may be only part of the outward expression of some disorder of the brain which itself produces testamentary incapacity, but so far as the aphasia itself is concerned it may be considered that everything depends upon the evidence first of understanding and second of power of expressing assent or the opposite with certainty.

In this case it would appear that the condition was one of "simple" motor aphasia, the power of word formation being interfered with, while the ingoing word-tracks, whether by eye or ear, were practically open. The matter then was fairly plain sailing when once it was proved that she was able to understand spoken and written language and to express assent.

Sir William Bateman, in his work on aphasia, has laid it down that "aphasia does not necessarily entail testamentary incapacity; in fact, wills or other legal documents should, as a rule, be recognised as valid when the parties understand fully what is put before them and can express assent or dissent with certainty, whether by articulate, written, or gesture language."

Perhaps we may add that even to those whose mind and power of expression are absolutely untouched it may be far from easy, in fact it may be impossible so to recall at the moment the names of all the persons to whom it may be wished to leave some token of regard. Wills, indeed, are probably but rarely perfect instruments, but, as Dr. Savage says, the present bent of an English jury is to find for any will, not in itself unreasonable, if it expresses the wishes of the testator approximately, hence the possibility of an omission, even though such omission might be owing to the method adopted, was not held to invalidate the will in view of the fact that when the will had been drawn up and read to her the testatrix had understood it and assented to it.

